# A peroxiredoxin 1–reactive oxygen species feedback loop modulates nuclear factor kappa B activity in osteoclastogenesis

**DOI:** 10.1186/s43556-026-00559-5

**Published:** 2026-08-27

**Authors:** Hyerim Lee, Sheunghun Lee, You-Jee Jang, Kyubin Lee, Seon Young Hwang, Goo Taeg Oh, Jae-Il Park, Sun-Ju Yi, Kyunghwan Kim

**Affiliations:** 1 Department of Biology, Chungbuk National University, Cheongju, Chungbuk Republic of Korea; 2 Department of Biomedical Laboratory Science, Honam University, Gwangju, Republic of Korea; 3 Department of Life Sciences, Ewha Womans University, Seoul, Republic of Korea; 4 Korea Basic Science Institute, Gwangju, Republic of Korea

**Keywords:** PRDX1, Reactive oxygen species, P65, Osteoclastogenesis, Osteoporosis

## Abstract

Osteoclastogenesis is driven by tightly coordinated transcriptional programs downstream of receptor activator of nuclear factor-κB ligand (RANKL) signaling, in which reactive oxygen species (ROS) function as essential secondary messengers. Although extracellular peroxiredoxin 1 (PRDX1) has been implicated in the suppression of osteoclast differentiation, the role of intracellular PRDX1 in regulating osteoclastogenic signaling remains poorly understood. Here, we identify intracellular PRDX1 as a redox-sensitive negative regulator of osteoclastogenesis and bone resorption. PRDX1 deficiency markedly enhanced osteoclast differentiation, bone resorption activity, and osteoporotic phenotypes in vivo. Transcriptomic and mechanistic analyses revealed that PRDX1 attenuates osteoclastogenic gene transcription by blocking the nuclear factor kappa B (NF-κB)/p65 signaling axis. Loss of PRDX1 increased intracellular ROS accumulation, promoted p65 nuclear translocation and promoter occupancy, thereby amplifying osteoclastogenic transcriptional programs. Mechanistically, PRDX1 underwent TNF receptor associated factor 6 (TRAF6)-mediated Lys67-dependent ubiquitylation and lysosomal degradation in response to RANKL signaling. In addition, RANKL-induced Src activation promoted phosphorylation of PRDX1 at Tyr194, which enhanced TRAF6-mediated ubiquitylation without substantially altering overall TRAF6 binding. Together, these findings reveal a PRDX1–ROS feedback loop that modulates NF-κB activity during osteoclastogenesis and identify regulated PRDX1 degradation as a mechanism by which RANKL signaling amplifies osteoclastogenic responses. These findings establish intracellular PRDX1 as an important modulator of skeletal homeostasis, suggesting its potential relevance as a pharmacological target in diseases characterized by excessive bone resorption.

**Supplementary Information:**

The online version contains supplementary material available at https://doi.org/10.1186/s43556-026-00559-5.

## Introduction

Reactive oxygen species (ROS) are oxygen-derived molecules that participate in diverse physiological and cellular signaling processes [1]. To maintain redox balance and prevent oxidative damage, cells have evolved a sophisticated antioxidant defense system composed of enzymes and molecules that neutralize excess ROS [2]. As such, maintaining redox homeostasis is critical for cellular processes including metabolism, growth, differentiation, and immune responses [3].

The generation of functional osteoclasts from precursor cells is governed by a coordinated intracellular signaling network. While macrophage colony-stimulating factor (M-CSF) drives the survival and expansion of osteoclasts precursor (OCP) populations, receptor activator of nuclear factor kappa B ligand (RANKL) delivers the definitive signal driving osteoclast differentiation. Upon binding to receptor activator of nuclear factor kappa B (RANK), RANKL activates downstream pathways, including nuclear factor kappa B (NF-κB) signaling, which promotes the expression of osteoclast-related genes such as nuclear factor of activated T cells 1 (Nfatc1), matrix metalloproteinase 9 (Mmp9), cathepsin K, and tartrate-resistant acid phosphatase (Trap). In addition, RANKL–RANK signaling triggers ROS generation, which function as crucial mediators of osteoclast differentiation and activation [4–8].

Antioxidants such as N-acetylcysteine (NAC) have been shown to inhibit RANKL-induced osteoclastogenesis by reducing ROS levels [6, 9, 10]. In addition, several antioxidant enzymes—including nuclear respiratory factor 1 (NRF1), NRF2, peroxiredoxin 1 (PRDX1), PRDX5, superoxide dismutase 2, and thioredoxin 1—have demonstrated inhibitory effects on osteoclast differentiation [11–15]. These findings underscore the critical role of ROS in regulating osteoclastogenesis [10, 16, 17]. In osteoporosis, bone resorption outpaces formation, disrupting the normal balance of bone remodeling. This leads to skeletal weakening and a heightened risk of fractures [18]. Emerging evidence suggests that oxidative stress—caused by excess ROS and impaired redox homeostasis—plays a key role in the progression of osteoporosis [10, 19].

PRDXs function as antioxidant enzymes that mitigate oxidative stress by reducing peroxide substrates [20]. There are six mammalian PRDXs, which are widely distributed across intracellular compartments. Mammalian PRDXs are divided into typical 2-Cys (PRDX1–4), atypical 2-Cys (PRDX5), and 1-Cys (PRDX6) groups on the basis of their catalytic cysteine chemistry [21, 22]. PRDX5 and PRDX6 have been implicated in the regulation of osteogenic differentiation [11, 23]. PRDX1 is implicated in diverse cellular processes, including tumor suppression, apoptosis, and molecular chaperone activities [24–28]. Our recent studies demonstrated that extracellular PRDX1 functions as a ligand for toll-like receptor 4 (TLR4) and suppresses osteoclast differentiation by modulating p65 signaling [29]. While extracellular PRDX1 has been implicated in TLR4-mediated suppression of osteoclastogenesis, the intracellular, enzymatic function of PRDX1 during RANKL-induced osteoclast differentiation has remained poorly understood. In particular, the reciprocal relationship between PRDX1 and ROS during osteoclast differentiation has not been well characterized.

Our study identifies PRDX1 as a redox-sensitive suppressor of osteoclastogenesis and bone resorption. PRDX1 inhibition or depletion significantly enhances osteoclast differentiation without affecting osteoclast precursor proliferation. Loss of *Prdx1* in mice was associated with reduced bone mass and increased osteoclast-mediated bone resorption, resulting in an osteoporotic phenotype. RNA-seq analysis identified osteoclast-related genes regulated by PRDX1, particularly through NF-κB signaling**,** with PRDX1 depletion enhancing p65 nuclear translocation and transcriptional activity. Mechanistically, we found that RANKL-mediated ROS signaling promotes TNF receptor associated factor 6 (TRAF6)-mediated ubiquitylation and lysosomal degradation of PRDX1, while Src-dependent phosphorylation of PRDX1 at Tyr 194 (Y194) appears to facilitate this process. Collectively, these findings establish PRDX1 as a negative regulator of osteoclastogenesis and reveal a PRDX1–ROS feedback mechanism that modulates NF-κB signaling during osteoclast differentiation.

## Results

### PRDX1 suppresses osteoclast differentiation and bone resorption

**Fig. 1:**
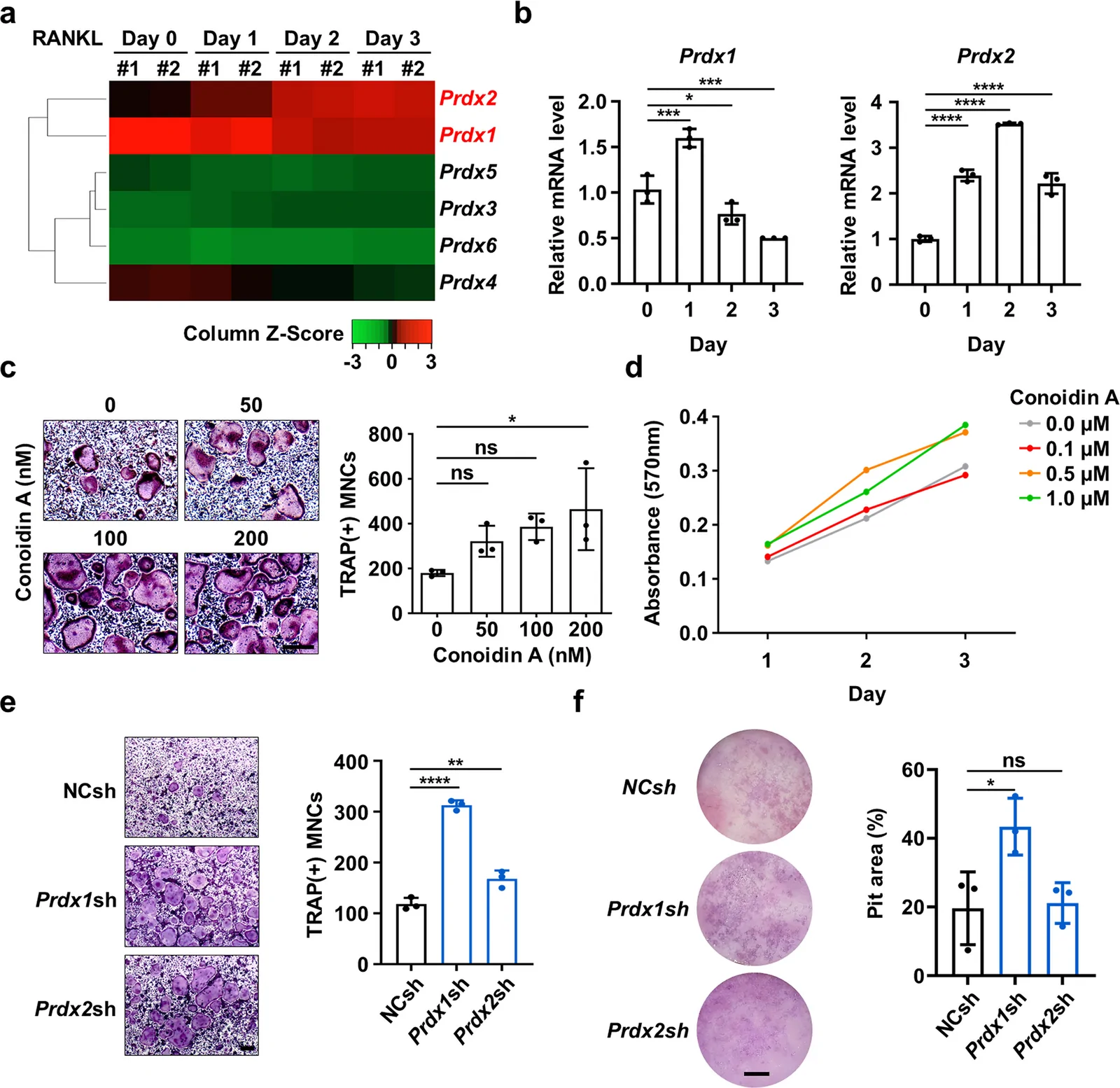
Peroxiredoxin 1** (**PRDX1) suppresses osteoclast differentiation. **a** Microarray profiling of *Prdx* mRNA expression during osteoclast differentiation. **b** Quantitative analysis of *Prdx1* and *Prdx2* mRNA levels during osteoclast differentiation. **c** Tartrate-resistant acid phosphatase (TRAP) staining of osteoclasts differentiated from osteoclasts precursor (OCP) cells treated with conoidin A. Scale bar, 200 μm. **d** Cell proliferation assay during osteoclastogenesis in the presence of conoidin A. **e** TRAP staining of OCP cells transfected with control shRNA, *Prdx1* shRNA or *Prdx2* shRNA. Scale bar, 200 μm. The data are represented by mean ± SD of three independent experiments. **f** Bone-resorption activity of osteoclasts derived from control shRNA, *Prdx1* shRNA or *Prdx2* shRNA. Scale bar, 1 mm. One-way ANOVA followed by Dunnett’s multiple comparisons test (**b**, **c**, **e**, **f**). *P < 0.05; **P < 0.01; ***P < 0.001; ****P < 0.0001

To identify PRDX family members involved in osteoclast differentiation, we examined the expression levels of *Prdx* genes using microarray analysis. Our results revealed that *Prdx1* and *Prdx2* showed higher expression levels than other PRDX family members during osteoclastogenesis (Fig. 1a). Notably, *Prdx1* expression initially increased at day 1 following RANKL treatment but gradually declined thereafter. In contrast, *Prdx2* expression progressively increased in response to RANKL stimulation. These dynamic expression patterns were further confirmed by RT-qPCR analysis (Fig. 1b). To determine the roles of PRDX1 and PRDX2 in osteoclast differentiation, OCPs were treated with conoidin A, a selective PRDX1/2 inhibitor. This treatment significantly increased osteoclast formation in a concentration-dependent manner, while OCP proliferation remained unchanged (Fig. 1c, d). Further investigation using shRNA-mediated knockdown showed that *Prdx1* depletion significantly increased osteoclast differentiation, whereas *Prdx2* depletion had a lesser effect on RANKL-mediated osteoclast differentiation (Figs. 1e and S1a). Because PRDX1 and PRDX2 are closely related members of the PRDX family, we further examined whether *Prdx1* depletion affected PRDX2 expression. However, neither PRDX2 mRNA nor protein levels were significantly altered by *Prdx1* knockdown (Fig. S1b, c). In addition, the expression levels of other *Prdx* family members were not noticeably changed following *Prdx1* depletion (Fig. S1d). These findings indicate that *Prdx1* depletion does not induce compensatory changes in the expression of other PRDX family members.

We further performed bone resorption assays to determine whether the enhanced osteoclast differentiation was accompanied by functional changes in osteoclast activity. Consistent with the differentiation results, *Prdx1* knockdown markedly increased the resorption pit area relative to the control group, whereas *Prdx2* knockdown showed little or no discernible impact (Fig. 1f). These findings further support a specific role for PRDX1 in suppressing osteoclast differentiation and bone resorption.

### PRDX1 deficiency causes osteoporotic bone loss in male mice

**Fig. 2:**
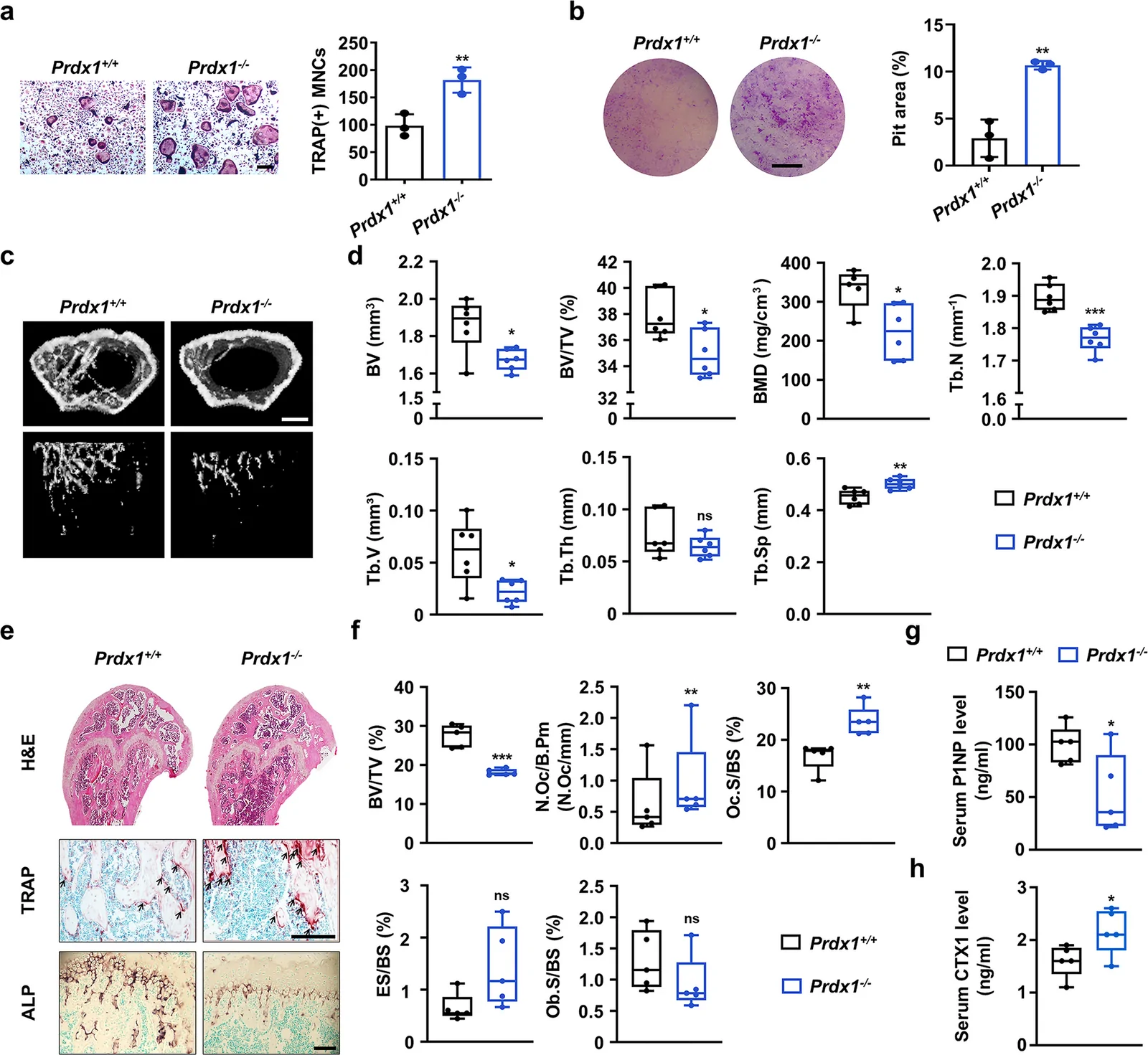
Deletion of PRDX1 elevates bone mass by suppressing osteoclast activity in vivo*.* **a** TRAP staining showing osteoclasts differentiated from wild-type (WT, *Prdx1*^+*/*+^) or knockout mice (KO, *Prdx1*^*−/−*^) OCP cells. Scale bar, 200 μm. **b** Bone-resorption activity of osteoclasts derived from *Prdx1*^+*/*+^ or *Prdx1*^*−/−*^ OCP cells. Scale bar, 1 mm. **c** Representative micro-CT image of proximal femurs from 3-month-old male mice (top, axial view; bottom, longitudinal view). Scale bar, 1 mm. **d** Quantitative micro-CT analysis of femurs from 3-month-old male *Prdx1*^+*/*+^ and *Prdx1*^*−/−*^ mice (*n* = 6). Bone volume (BV), trabecular bone volume per tissue volume (BV/TV), bone mineral density (BMD), trabecular number (Tb.N), trabecular volume (Tb.V), and trabecular thickness (Tb.Th), trabecular separation (Tb.Sp) **e**, **f** H&E, TRAP, and ALP staining were performed under the same conditions as in **c** (*n* = 6). Scale bar, 100 μm. TRAP-positive osteoclasts are denoted by black arrows. H&E, Hematoxylin and eosin; ALP, alkaline phosphatase; N. Oc/B.Pm, osteoclast number per bone perimeter; Oc.S/BS, osteoclast surface per bone surface; ES/BS, eroded surface per bone surface; Ob. S/BS, osteoblast surface per bone surface. **g**, **h** Serum levels of bone turnover markers, including procollagen type I N-terminal propeptide (P1NP) (**g**) and C-terminal telopeptide of type I collagen (CTX-I) (**h**), measured in *Prdx1*^+*/*+^ and *Prdx1*^*−/−*^mice. In **a** and **b**, the data are represented as mean ± SD of three independent experiments. Two-tailed *t*-test (**a**, **b**, **d**, **f**) and unpaired *t*-test (**g**, **h**). *P < 0.05; **P < 0.01; ***P < 0.001; ns, not significant

Based on these in vitro findings, we next examined the consequences of PRDX1 deficiency using OCPs and age-matched male *Prdx1* knockout mice (*Prdx1⁻/⁻*). As expected, the loss of PRDX1 increased osteoclast differentiation (Figs. 2a and S2). Moreover, *Prdx1* deletion in osteoclasts enhanced bone resorption, with *Prdx1*^*−/−*^ OCPs exhibiting a greater resorption pit area compared with OCPs from the wild-type mice (*Prdx1*^+*/*+^) (Fig. 2b). To determine the biological significance of PRDX1 in vivo, we analyzed bone microarchitecture in three-month-old homozygous *Prdx1*^+*/*+^ and *Prdx1*^*−/−*^ mice. Relative to the control group, the latter group exhibited substantial bone loss. This was reflected by reduced bone volume (BV), trabecular bone volume per tissue volume (BV/TV), bone mineral density (BMD), trabecular number (Tb.N), trabecular volume (Tb.V), and trabecular thickness (Tb.Th), accompanied by an increase in trabecular separation (Tb.Sp) (Fig. 2c, d). To assess the effects of PRDX1 deficiency on bone remodeling, femoral sections from *Prdx1*^+^*/*^+^ and *Prdx1*^-^*/*^-^ mice were subjected to TRAP and alkaline phosphatase (ALP) staining. Consistent with enhanced osteoclastogenesis, *Prdx1*^-^*/*^-^ femurs contained more TRAP-positive osteoclasts than wild-type femurs (Fig. 2e). This was further validated by histomorphometric analysis, which showed marked elevation in trabecular osteoclast number (N.Oc/B.Pm), osteoclast surface per bone surface (Oc.S/BS), and eroded surface per bone surface (ES/BS) in *Prdx1*^-^*/*^-^ mice. In contrast, ALP-positive cell number, osteoblast surface per bone surface (Ob.S/BS), and trabecular bone volume per tissue volume (BV/TV) tended to be lower in *Prdx1*^-^/^-^ mice, although the reduction in Ob.S/BS did not reach statistical significance (Fig. 2f). Consistent with these histological findings, serum analysis showed reduced levels of procollagen type I N-terminal propeptide (P1NP) and increased levels of C-terminal telopeptide of type I collagen (CTX-I) in *Prdx1*^*-*/*-*^ mice (Fig. 2g, h). Collectively, these findings suggest that *Prdx1* deficiency drives an osteoporosis phenotype associated with increased osteoclast function and reduced bone formation.

### PRDX1 restrains osteoclastogenic gene expression and reactive oxygen species accumulation

**Fig. 3:**
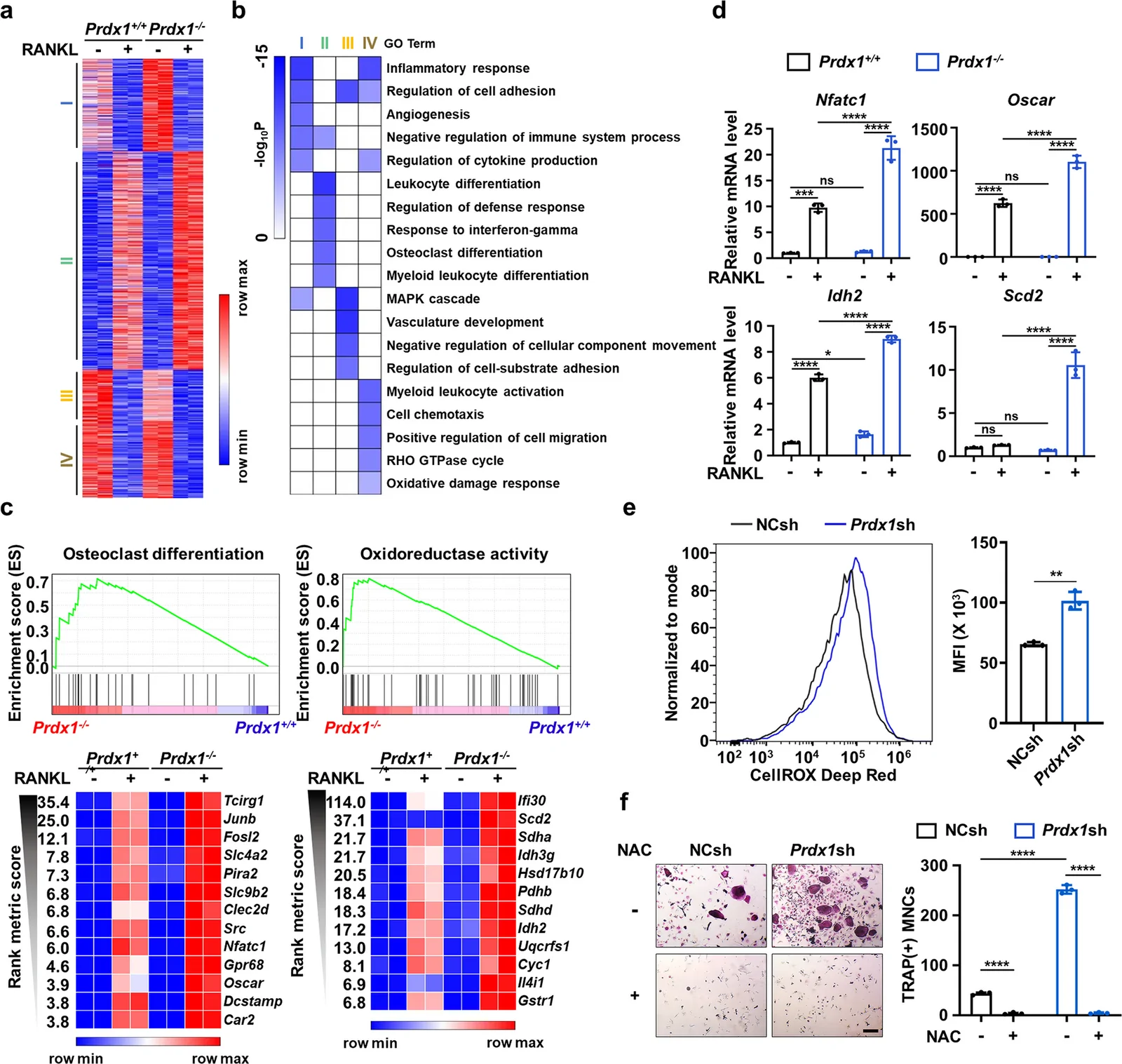
PRDX1 modulates the transcriptomic profile during RANKL-induced osteoclast differentiation. **a** K-means (K = 4) clustering of 1,731 differentially expressed genes from pairwise comparisons between OCPs from *Prdx1* WT and KO mice, with or without RANKL stimulation. **b** Heatmap showing the *p*-value significance of the gene ontology (GO) term enrichment for genes in each cluster. **c** Gene set enrichment analysis of 1,731 genes as in **a** reveals significant enrichment of pathways involved in osteoclast differentiation and oxidoreductase activity. **d** Relative mRNA levels of representative genes (**c**) were validated via qRT-PCR. **e** Flow cytometry histograms showing cellular ROS levels in OCP cells transduced with either control shRNA or *Prdx1* shRNA and stimulated with RANKL (100 ng/ml, 10 min). Bar graphs represent the median fluorescence intensity (MFI). **f** TRAP staining of OCP cells expressing either control shRNA or *Prdx1* shRNA with or without N-acetyl cysteine (NAC) (10 mM). Scale bar, 200 μm. Data are represented as mean ± SD of three independent experiments. Statistical analyses were performed using two-way ANOVA followed by Tukey’s multiple comparisons test in (**d**), two-tailed *t*-test (**e**), and two-way ANOVA followed by Šídák's multiple comparisons test (**f**). **P < 0.01; ***P < 0.001; ****P < 0.0001; ns, not significant

To investigate how PRDX1 regulates osteoclastogenesis, we identified PRDX1-regulated genes and analyzed their functions. RNA-seq was performed using OCPs from *Prdx1*^+*/*+^ and *Prdx1*^*−/−*^ mice, with or without RANKL treatment. Four clusters of 1,731 differentially expressed genes (DEGs) were analyzed by gene ontology (GO) enrichment analysis (Fig. 3a, b). Following RANKL stimulation, Cluster II genes associated with osteoclast differentiation exhibited more pronounced upregulation in *Prdx1*^*−/−*^ cells than in their *Prdx1*^+*/*+^ counterparts. Furthermore, gene set enrichment analysis (GSEA) revealed that key pathways encompassing osteoclast differentiation and oxidoreductase activity were markedly enhanced in the absence of PRDX1 under RANKL stimulation (Fig. 3c). Specifically, pro-osteoclastogenic genes such as *Nfatc1* and *osteoclast-associated receptor* (*Oscar*), as well as oxidoreductase-related genes including *isocitrate dehydrogenase 2* (*Idh2*) and stearoyl-CoA desaturase 2 (*Scd2*), were significantly upregulated in *Prdx1⁻/⁻* OCPs. These transcriptomic changes were validated by quantitative RT-qPCR analysis (Fig. 3d). ROS levels were substantially increased in *Prdx*1⁻/⁻ cells after RANKL treatment (Fig. 3e). Moreover, NAC treatment significantly inhibited RANKL-induced osteoclast formation in wild-type OCPs, with an even greater suppressive effect observed in *Prdx1*-deficient OCPs (Fig. 3f). These findings highlight the central role of PRDX1 in modulating ROS levels and regulating the expression of genes critical for osteoclastogenesis.

### PRDX1 negatively regulates NF-ĸB transactivity

**Fig. 4:**
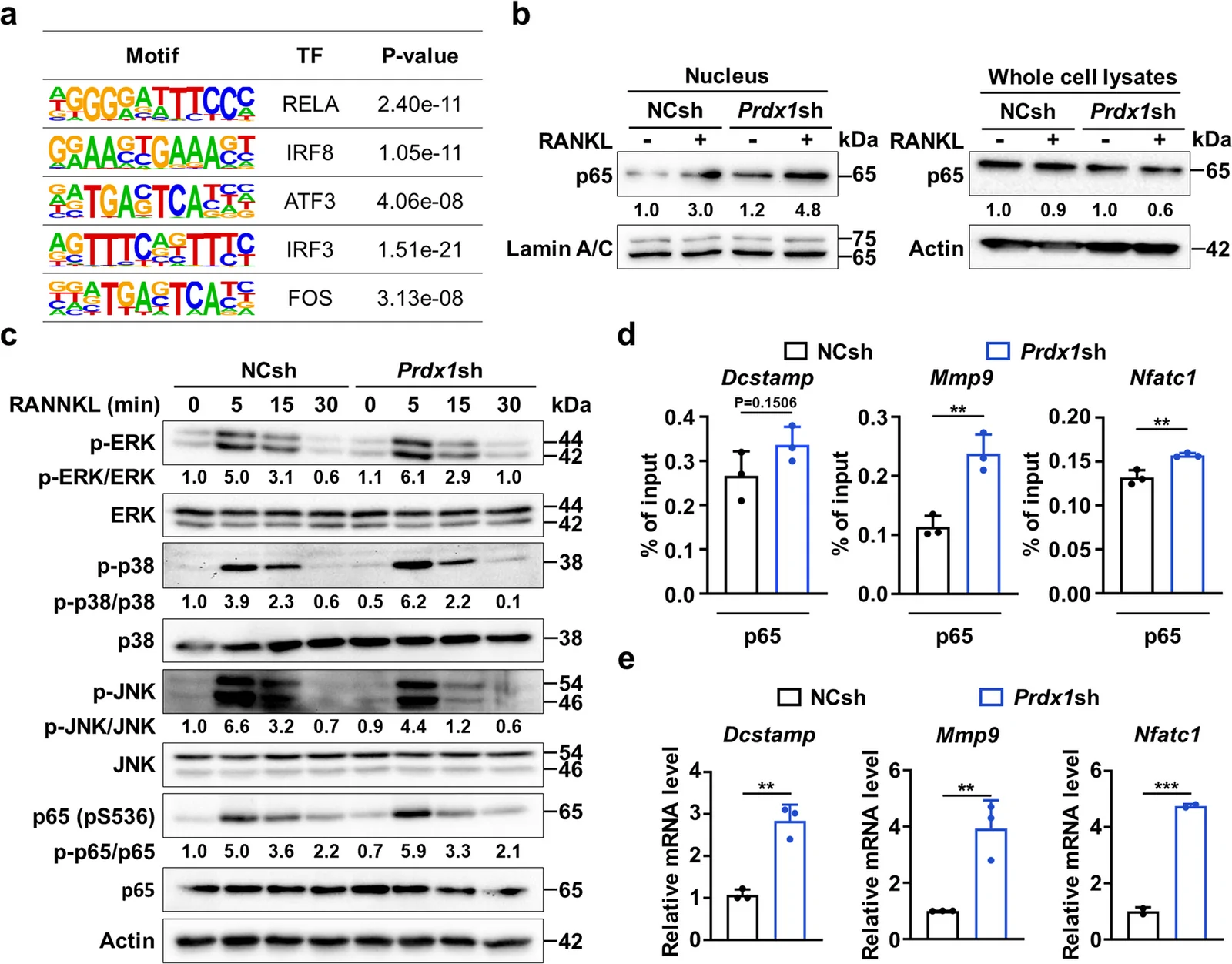
PRDX1 acts as a negative regulator of the p65 signaling axis. **a** Key transcription factor motifs significantly enriched in PRDX1-associated genes were identified by HOMER de novo motif analysis. **b** Nuclear and whole cell lysates from osteoclast precursor cells transduced with control or *Prdx1* shRNA were analyzed following RANKL stimulation (100 ng/ml, 30 min). Nuclear translocation of p65 was assessed by Western blot. Band intensities were quantified using ImageJ and expressed as relative values normalized to Lamin A/C (nuclear) or Actin (whole cell). **c** Whole cell lysates from RANKL-stimulated OCPs (100 ng/ml) transduced with control or *Prdx1* shRNA were analyzed by Western blot for components of the NF-κB and MAPK signaling pathways. Band intensities were quantified using ImageJ and normalized to total protein levels or actin. Representative blots of two independent experiments. **d** ChIP assay showing p65 binding to *Dcstamp, Mmp9*, and *Nfatc1* promoters in *Prdx1*-depleted cells following RANKL treatment for 30 min. Data represent the mean ± SD of three independent experiments. Two-tailed *t*-test, **P < 0.01. **e** Relative mRNA expression of *Dcstamp*, *Mmp9* and *Nfatc1* in mock- or *Prdx1*-depleted OCP cells treated with RANKL. The data are represented as mean ± SD of three independent experiments. Two-tailed *t*-test, **P < 0.01; ***P < 0.001

Next, we sought to identify transcription factors associated with PRDX1 during osteoclastogenesis. Motif analysis of Cluster II genes from Fig. 3a revealed that several transcription factors, including p65 (RELA), interferon regulatory factor 8 (IRF8), and activating transcription factor 3 (ATF3), were among the top-ranked candidates (Fig. 4a). Given the essential role of NF-κB in osteoclast differentiation [30–33], we investigated whether PRDX1 modulates the p65 signaling pathway. OCPs expressing control or *Prdx1*-targeting constructs were exposed to RANKL, after which p65 nuclear accumulation was evaluated by Western blotting. PRDX1 depletion led to enhanced nuclear localization of p65, while total p65 protein levels remained unchanged (Fig. 4b). In addition, PRDX1 knockdown apparently increased RANKL-mediated extracellular signal-regulated kinase (ERK), p38, and p65 phosphorylation (Fig. 4c), indicating that PRDX1 negatively regulates multiple signaling pathways that promote osteoclast differentiation.

To further elucidate the role of PRDX1 in p65-mediated transcriptional regulation, chromatin immunoprecipitation (ChIP) assays were performed using a p65 antibody. In response to RANKL stimulation, p65 occupancy at target gene promoter regions was observed, and this binding was significantly increased upon PRDX1 depletion (Fig. 4d). The enhanced promoter binding correlated with elevated expression of the corresponding target genes (Fig. 4e). Given its antioxidant role, PRDX1 appears to negatively regulates RANKL-induced NF-κB signaling, at least in part, through modulation of intracellular ROS levels, thereby limiting p65 nuclear activity and target gene transcription during osteoclastogenesis.

### Reactive oxygen species promote PRDX1 ubiquitylation and lysosomal degradation

**Fig. 5:**
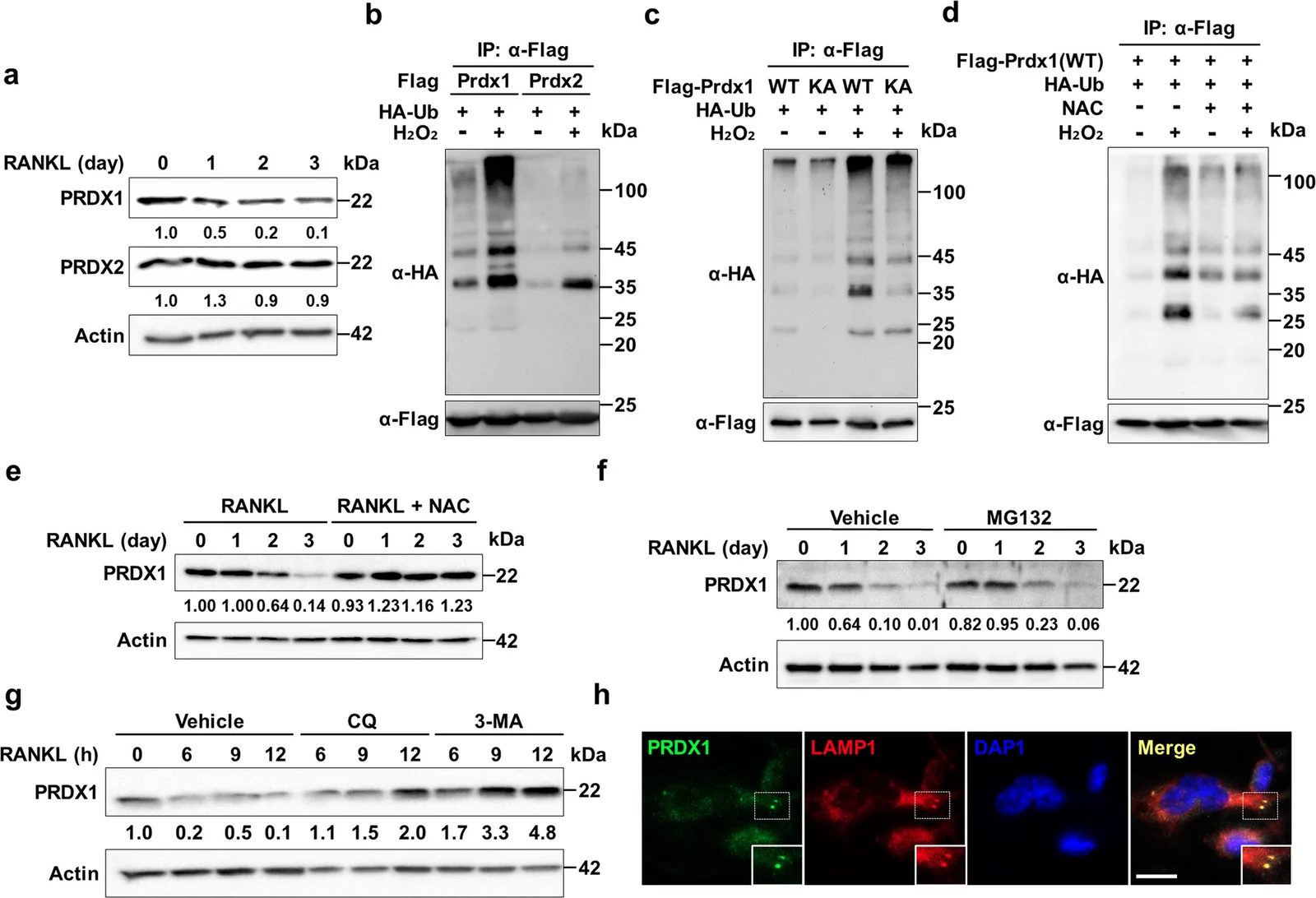
RANKL-induced ROS regulate PRDX1 stability by promoting its lysosomal degradation. **a** Expression levels of PRDX1 and PRDX2 in OCP cells during osteoclast differentiation were analyzed by Western blotting. Band intensities were quantified using ImageJ and normalized to Actin. **b** 293 T cells transfected with Flag-*Prdx1* or Flag-*Prdx2* together with HA-Ub were treated with 1 mM H₂O₂ for 1 h. Cell lysates were immunoprecipitated with anti-Flag antibody, and immunocomplex were separated and detected with Western analysis using anti-HA and anti-Flag antibody. **c** 293 T cells transfected with Flag-*Prdx1* wild type or Flag-*Prdx1* K67A (KA) along with HA-Ub were treated with 1 mM H₂O₂ for 1 h. Immunoprecipitation and Western blotting were performed as in **b**. **d** 293 T cells transfected with Flag-*Prdx1* wild type and HA-Ub were exposed to 1 mM H₂O₂ for 1 h with or without NAC (10 mM). Immunoprecipitation and Western blotting were performed as described in **b**.** e** Western blot analysis of PRDX1 expression during osteoclast differentiation with or without NAC (10 mM). Band intensities were quantified using ImageJ and normalized to Actin. **f** Whole cell lysates during osteoclast differentiation derived from OCP cells in the absence or presence of MG132 (10 μM) were subjected to Western blotting. Band intensities were quantified using ImageJ and normalized Actin. **g** OCP cells were stimulated with RANKL for 36 h, followed by treatment with chloroquine (CQ, 10 μM) or 3-methyladenine (3-MA, 5 mM) in fresh differentiation media for 6, 9, or 12 h. PRDX1 expression levels were analyzed by Western blotting. Band intensities were quantified using ImageJ and normalized to actin. **h** OCP cells were stimulated with RANKL for 48 h and stained for PRDX1 and the lysosomal marker LAMP1, followed by confocal microscopy analysis. Nuclei were counterstained with DAPI. Representative images demonstrating colocalization of PRDX1 with LAMP1 are shown. Scale bar, 10 μm

Although RANKL-induced osteoclast differentiation is tightly regulated by various negative regulators, RANKL signaling ultimately promotes differentiation by modulating these inhibitory factors. Our study identified PRDX1 as a negative regulator of RANKL signaling, raising the question of how RANKL modulates PRDX1 function. To address this, we examined PRDX1 protein levels following RANKL treatment. A marked decrease in PRDX1 protein levels was observed after two days of RANKL stimulation, while PRDX1 mRNA levels showed only a modest reduction (Figs. 1b and 5a). These results suggest that PRDX1 expression is predominantly regulated at the translational or post-translational level, with additional control at the transcriptional level. The observed reduction in PRDX1 protein abundance is in line with evidence that oxidative conditions trigger PRDX1 polyubiquitylation and degradation by proteasomal and lysosomal mechanisms [34]. To investigate the mechanisms underlying PRDX1 degradation, we introduced hemagglutinin-tagged ubiquitin into 293 T cells expressing Flag-PRDX1 and immunoprecipitated ubiquitinated PRDX1 with or without 1 mM H_2_O_2_ treatment. Exposure to H_2_O_2_ markedly increased PRDX1 polyubiquitination (Fig. 5b, lane 1 versus lane 2). In contrast, PRDX2 ubiquitination was only modestly enhanced by H_2_O_2_ treatment (lane 3 versus lane 4). Furthermore, protein expression levels of PRDX2 remained unchanged following RANKL treatment (Fig. 5a), suggesting differential regulation for PRDX1 and PRDX2 during RANKL-induced ROS signalling.

To identify potential ubiquitylation sites responsible for this selective regulation, we integrated predictions from the ubiquitylation site prediction tool RUBI 1.0 (http://old.protein.bio.unipd.it/rubi/) with sequence alignment between PRDX1 and PRDX2. This analysis identified Lys67 (K67) as a putative ubiquitylation site in PRDX1 (Fig. S3). Notably, H_2_O_2_-induced ubiquitylation was markedly increased in wild-type PRDX1, whereas the K67A mutant exhibited significantly reduced ubiquitylation (Fig. 5c), suggesting that K67 is a key residue targeted for ubiquitylation in response to RANKL-mediated ROS signaling. Since RANKL-induced ROS appeared to drive PRDX1 degradation, we tested whether ROS scavenging could rescue PRDX1 expression. Treatment with NAC effectively reduced H_2_O_2_-induced ubiquitylation of PRDX1 (Fig. 5d) and restored PRDX1 protein expression (Fig. 5e).

To further determine the degradation pathway involving PRDX1, we initially focused on proteasome-mediated degradation. Surprisingly, treatment with the proteasome inhibitor, MG132, did not protect PRDX1 from degradation induced by RANKL (Fig. 5f). Next, we examined the involvement of lysosomal degradation in PRDX1 stability. After 36 h of RANKL priming, OCPs were cultured for a further 6, 9, or 12 h in fresh differentiation medium containing chloroquine (CQ) or 3-methyladenine (3-MA), which block lysosomal degradation. Remarkably, treatment with both CQ and 3-MA effectively prevented PRDX1 degradation (Fig. 5g). Consistent with these findings, confocal microscopy analysis revealed substantial colocalization of PRDX1 with the lysosomal marker lysosome-associated membrane glycoprotein 1 (LAMP1) following RANKL stimulation (Fig. 5h). These results suggest that the lysosomal degradation pathway plays a critical role in regulating PRDX1 stability during osteoclastogenesis.

### TRAF6 and Src-dependent PRDX1 phosphorylation promote PRDX1 ubiquitylation

**Fig. 6:**
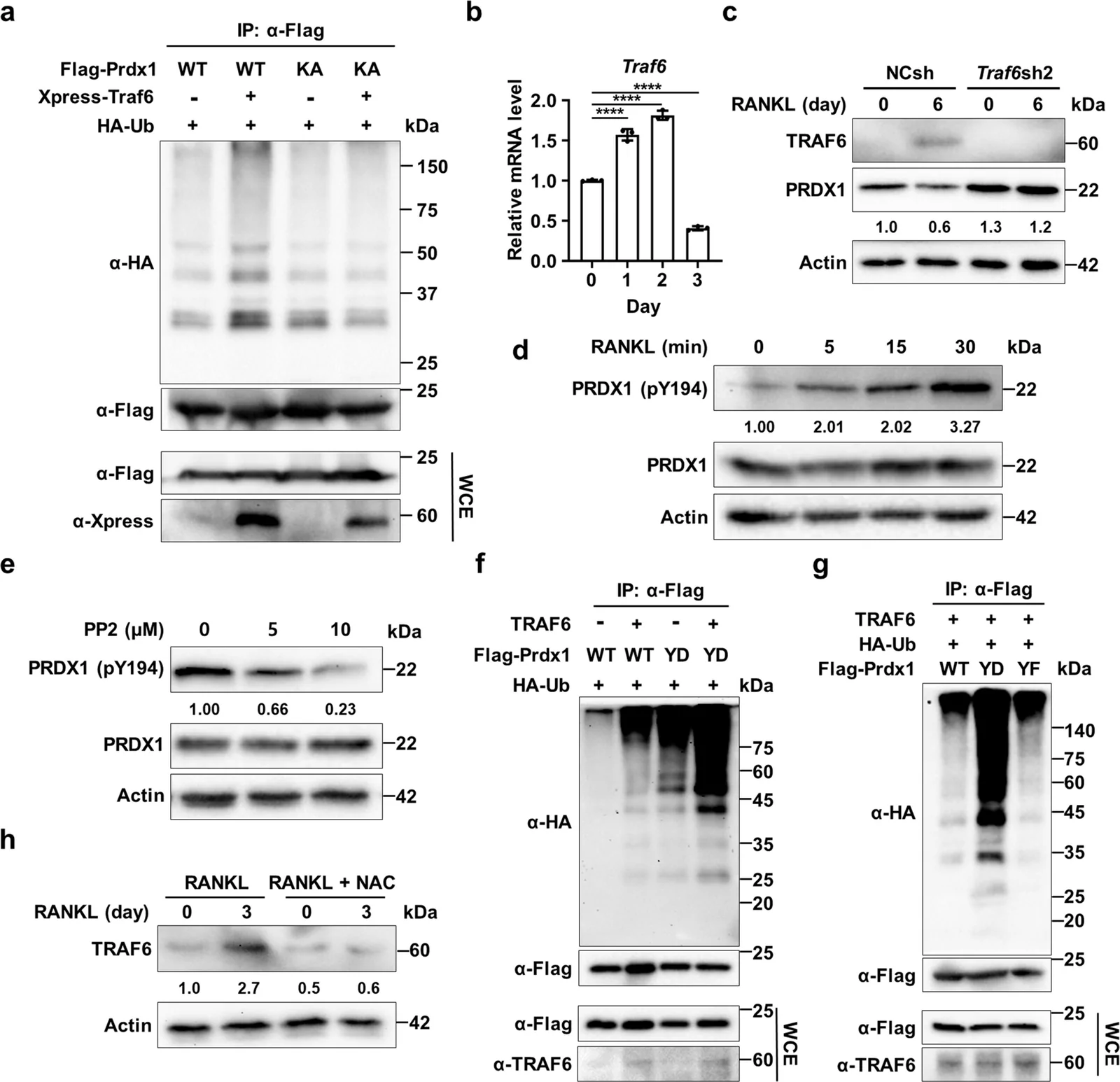
TRAF6-mediated ubiquitylation of PRDX1 is facilitated by Y194 phosphorylation. **a** 293 T cells were transfected with Flag-*Prdx1* wild-type (WT) or Flag-*Prdx1* K67A (KA) together with HA-Ub, in the presence or absence of Xpress-*Traf6*. Cell lysates were immunoprecipitated with anti-Flag antibody and analyzed by Western blotting with anti-Xpress antibody. **b** mRNA expression levels of *Traf6* upon RANKL treatment. **c** Cell lysates from mock- or *Traf6*-depleted OCPs were analysed by Western blotting using antibodies against TRAF6, PRDX1, and Actin. **d** OCPs were stimulated with RANKL (100 ng/ml) for the indicated times (0, 5, 15, and 30 min). Whole-cell lysates were prepared and analyzed by Western blotting using the indicated antibodies. **e** OCPs were pre-treated with the Src inhibitor PP2 (5 or 10 μM) for 1 h and subsequently stimulated with RANKL (100 ng/ml) for 15 min. Whole-cell lysates were analyzed by Western blotting using the indicated antibodies. **f**,** g** 293 T cells were transfected with Flag-Prdx1 wild-type (WT), Y194D (YD), or Y194F (YF) together with Xpress-Traf6 and HA-Ub, as indicated. Cell lysates were subjected to immunoprecipitation and Western blot analysis as described in **a** to assess PRDX1 ubiquitylation. **h** TRAF6 expressions were assessed during osteoclast differentiation with or without NAC (10 mM). Band intensities were quantified using ImageJ and normalized to Actin (*n* = 3). Data are mean ± SD. One-way ANOVA followed by Dunnett’s multiple comparisons test determined significance (**P < 0.01; ****P < 0.0001)

Given recent studies demonstrating that PRDX1 physically interacts with TRAF6, a lysosome-associated E3 ubiquitin ligase, and can modulate its enzymatic activity [35], we next investigated whether TRAF6 reciprocally regulates PRDX1 stability through ubiquitylation. Consistent with this possibility, TRAF6 markedly promoted PRDX1 ubiquitylation at K67 in 293 T cells, as confirmed by co-immunoprecipitation and ubiquitin-conjugation assays (Fig. 6a). Importantly, RANKL stimulation not only enhanced PRDX1 ubiquitylation but also increased TRAF6 expression levels (Fig. 6b, c), suggesting that TRAF6 serves as a key mediator linking RANKL signaling to PRDX1 degradation. To further determine the functional role of this regulatory axis, we investigated the effect of *Traf6* depletion. Consistent with previous reports [36, 37] demonstrating the essential role of TRAF6 in osteoclast differentiation, loss of *Traf6* markedly reduced RANKL-induced osteoclastogenesis (Fig. S4a). Notably, *Traf6* depletion also resulted in robust stabilization of PRDX1 protein during osteoclast differentiation (Fig. 6c). These findings indicate that TRAF6-dependent ubiquitylation is a critical determinant of PRDX1 turnover and osteoclastogenic potential.

Previous studies have suggested that phosphorylation of PRDX1 at Y194 by Src kinase modulates its interaction with binding partners [38]. Since RANKL signaling is known to activate Src kinase, we examined whether PRDX1 undergoes phosphorylation at Y194 during osteoclastogenesis. As shown in Fig. 6d, RANKL treatment markedly increased phosphorylation of PRDX1 at Y194, which was substantially attenuated by the Src inhibitor PP2 (Fig. 6e), indicating that RANKL-induced Src activation mediates PRDX1 Y194 phosphorylation. We next investigated whether Y194 phosphorylation affects TRAF6-mediated ubiquitylation of PRDX1. Interestingly, the phosphomimetic mutant PRDX1-Y194D exhibited substantially enhanced TRAF6-mediated ubiquitylation compared with wild-type PRDX1, whereas the non-phosphorylatable mutant PRDX1-Y194F displayed ubiquitylation levels comparable to those of wild-type PRDX1 (Fig. 6f, g). However, co-immunoprecipitation analysis showed no major difference in the overall interaction between TRAF6 and PRDX1-WT, PRDX1-Y194D, and PRDX1-Y194F (Fig. S4b, c). These findings suggest that Y194 phosphorylation facilitates TRAF6-dependent ubiquitylation of PRDX1 without markedly altering the overall interaction between PRDX1 and TRAF6.

**Fig. 7:**
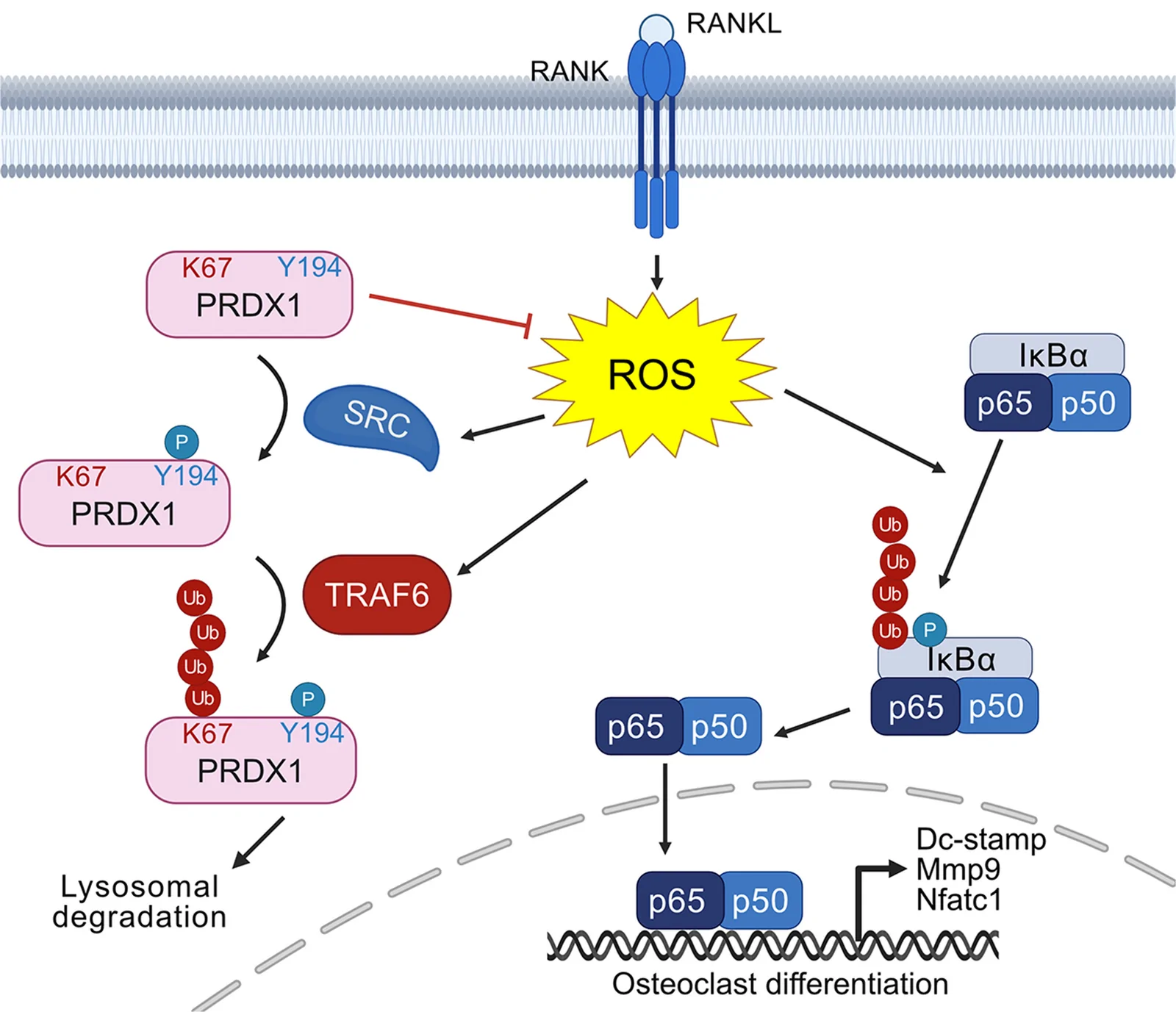
Proposed model of the redox-sensitive regulatory mechanism controlling osteoclastogenesis. RANKL-induced ROS increases TRAF6 expression, promoting K67-dependent ubiquitylation and lysosomal degradation of PRDX1. In parallel, RANKL-induced Src activation promotes phosphorylation of PRDX1 at Y194, which facilitates TRAF6-mediated ubiquitylation. Reduced PRDX1 levels lead to increased intracellular ROS accumulation and enhanced NF-κB/p65 activation, thereby amplifying osteoclastogenic signaling. Conversely, PRDX1 limits ROS accumulation and NF-κB activity, establishing a redox-sensitive regulatory circuit that controls osteoclast differentiation

Moreover, treatment with NAC efficiently suppressed RANKL-induced TRAF6 expression (Fig. 6h), highlighting the role of ROS as an upstream signal that enhances TRAF6 expression. Taken together, our findings suggest that RANKL signaling promotes PRDX1 degradation through coordinated ROS-dependent TRAF6 induction and Src-mediated Y194 phosphorylation, thereby relieving PRDX1-dependent suppression of osteoclastogenic signaling (Fig. 7).

## Discussion

Although previous studies have emphasized the role of ROS in promoting osteoclast differentiation downstream of RANKL [4, 5, 9, 12], the underlying mechanisms have remained unclear. Here, we identify PRDX1 as a key determinant regulator of osteoclastogenesis that operates via redox regulation and downstream NF-κB signaling axis. Our model suggests that the reciprocal regulation between ROS and PRDX1 fine-tunes ROS-mediated signaling, thereby contributing to the maintenance of bone homeostasis.

We found that among the six mammalian PRDXs, PRDX1 was predominantly expressed in osteoclast precursors, and its deficiency led to enhanced osteoclast formation in vitro and in vivo. The observation that *Prdx1*⁻/⁻ mice exhibit osteoporotic phenotypes due to excessive osteoclast activity further underscores the physiological relevance of this regulatory mechanism. Mechanistically, transcriptomic analyses revealed that PRDX1 regulates a set of osteoclast-related genes, including *Nfatc1*, *dendritic cell-specific transmembrane protein* (*Dcstamp*), and *Mmp9*, which are essential for differentiation and function. PRDX1 depletion increased the expression of these genes, supporting a role for PRDX1 as a negative regulator of osteoclastogenic transcriptional programs.

We further demonstrated that PRDX1 restrains NF-κB-dependent transcriptional activity, particularly targeting the p65 subunit, which plays a pivotal role in RANKL-induced osteoclastogenesis. In the absence of PRDX1, both nuclear translocation and promoter binding of p65 were markedly increased. Additionally, PRDX1 depletion enhanced the phosphorylation of p65 at Ser536, a modification known to promote its DNA binding and transcriptional activity. Given that PRDX1 loss also enhances the phosphorylation of ERK and p38 kinases known to promote p65 activation [39, 40], these findings suggest that PRDX1 may suppress p65 transactivation both directly and indirectly by modulating ERK and p38 signaling pathways.

In earlier work, we revealed that extracellular PRDX1 can act as a ligand for TLR4, hijacking p65 away from the RANKL signaling axis and thereby suppressing *Nfatc1* expression [29]. Similarly, *Wang *et al*.* demonstrated that recombinant PRDX1 protein inhibits osteoclastogenesis by reducing ROS levels and suppressing NFATc1 expression [41]. In contrast, the present study focuses on the intracellular functions of PRDX1, uncovering its mechanistic role in RANKL-induced osteoclast differentiation.

Crucially, our findings reveal that PRDX1 itself is post-translationally regulated by RANKL-induced ROS. We show that PRDX1 undergoes polyubiquitylation and degradation upon RANKL stimulation, particularly via lysosomal degradation. In contrast, PRDX2 remains largely unaffected. This raises an intriguing question about the underlying mechanism that contributes to the specific lysosomal degradation of PRDX1, but not PRDX2. Using a ubiquitylation prediction tool and sequence alignment, we identified K67 as a putative ubiquitylation site in PRDX1. Notably, this site is replaced by an arginine residue in PRDX2, which may contribute to the differential ubiquitylation profiles between the two proteins. Supporting this, mutation of K67 to alanine in PRDX1 markedly reduced its ubiquitylation, suggesting K67 as a critical residue for this modification.

Previous studies have shown that oxidation of cysteine residues can lead to protein misfolding and inactivation, marking them for degradation [34]. PRDX1 uniquely contains Cys83 (C83), which is absent in PRDX2 (Fig. S3) and has been implicated in hyperoxidation and oligomerization [42]. We hypothesized that oxidation of C83 might induce PRDX1 misfolding and subsequent ubiquitylation. However, substitution of Cys83 with Ser (C83S) did not affect ROS-mediated PRDX1 ubiquitylation (Fig. S5a), indicating that PRDX1 ubiquitylation may not be directly dependent on oxidation of this residue. Instead, we found that RANKL-induced ROS increased TRAF6 expression, leading to PRDX1 ubiquitylation and degradation.

In parallel, Sun et al*.* recently reported that OUT domain-containing protein 1 (OTUD1) stabilizes PRDX1 by deubiquitylating Lys16 (K16), thereby inhibiting osteoclast differentiation [43]. This suggests that PRDX1 stability is regulated by multiple ubiquitylation sites—K16 and K67—that are differentially targeted by distinct enzymatic systems. Whereas our study identifies TRAF6 as the E3 ligase for K67, it remains unclear whether TRAF6 also ubiquitylates K16, or whether a different E3 ligase is responsible for this modification. Likewise, although OTUD1 has been shown to remove ubiquitin from K16, it is not known whether OTUD1 can also deubiquitylate K67. Further studies will be required to resolve these questions.

Previous studies have suggested that PRDX1 can bind to TRAF6 and inhibit its E3 ligase activity [35], raising the question of how TRAF6 could simultaneously mediate PRDX1 ubiquitylation and degradation. We initially hypothesized that ROS-induced oxidation of PRDX1 might dissociate it from TRAF6; however, H₂O₂ treatment did not disrupt their interaction (Fig. S5b). Instead, we found that RANKL-induced Src activation promoted phosphorylation of PRDX1 at Y194, and that the Y194D mutant, but not Y194F, exhibited enhanced TRAF6-mediated ubiquitylation without a major change in overall TRAF6 binding.

These findings suggest that Y194 phosphorylation may not simply regulate the binding affinity between PRDX1 and TRAF6, but rather shift the PRDX1–TRAF6 complex toward a ubiquitination-permissive state. Thus, coordinated ROS-dependent TRAF6 induction and Src-mediated PRDX1 phosphorylation may promote PRDX1 degradation during osteoclastogenesis.

A limitation of this study is that the in vivo skeletal analyses were performed only in age-matched male mice. Because sex hormones, including estrogen, substantially influence osteoclast activity, bone remodeling, and osteoporosis-related phenotypes, the effects of PRDX1 deficiency may differ between male and female skeletons. Future studies using female mice, ideally across physiologically relevant hormonal states, will be required to define the full sex-dependent contribution of PRDX1 to skeletal development and bone homeostasis. In addition, although Y194 phosphorylation enhanced TRAF6-mediated PRDX1 ubiquitylation, the underlying structural mechanism remains unclear. Future studies should determine whether Y194 phosphorylation alters PRDX1 oligomerization, subcellular localization, or the functional configuration of the PRDX1–TRAF6 complex.

In summary, our study identifies PRDX1 as a redox-sensitive negative regulator of RANKL-induced osteoclastogenesis and bone resorption (Fig. 7). By restraining NF-κB/p65 transactivation and responding to intracellular ROS levels, PRDX1 acts as a key homeostatic regulator that prevents excessive osteoclast differentiation. Importantly, we found that RANKL signaling promotes TRAF6-mediated K67-dependent ubiquitylation and degradation of PRDX1 in a ROS-dependent manner. Our findings further suggest that Src-mediated phosphorylation of PRDX1 at Y194 facilitates this process without substantially altering overall TRAF6 binding, potentially shifting the PRDX1–TRAF6 complex toward a state permissive for ubiquitylation. Through this mechanism, RANKL signaling attenuates PRDX1 stability, thereby relieving PRDX1-mediated suppression of NF-κB activity and amplifying osteoclastogenic signaling. Collectively, these findings reveal a PRDX1–ROS feedback loop that fine-tunes NF-κB signaling during osteoclastogenesis and highlight regulated PRDX1 degradation as an important mechanism controlling bone remodeling.

## Materials and methods

### Microarray analysis

To evaluate changes in *Prdx* gene expression during RANKL-induced osteoclastogenesis, we analyzed publicly available microarray data (GSE57468) using the ArrayPipe software.

### Mice

*Prdx1* knockout mice were maintained on the C57BL/6 J background after thorough backcrossing [44]. Mice were kept in a temperature-regulated facility maintained at 22 °C under a 12-h light/12-h dark photoperiod. All experiments were conducted using age-matched male mice.

### Micro-CT analysis and Bone histomorphometry

Micro-CT and bone histomorphometry (n = 6) were performed on 3-month-old male *Prdx1*^+*/*+^ and *Prdx1*^*−/−*^ mice as previously described [45, 46].

### Osteoclast differentiation and pit formation assays

OCP cells were obtained based on a previously reported protocol [47]. For osteoclast differentiation, OCPs were cultured in 48-well plates with 30 ng/mL M-CSF (IKIM Bio, South Korea) and 100 ng/mL RANKL (IKIM Bio, South Korea). Following 4–6 days of differentiation, cells were fixed and subjected to TRAP staining. TRAP-positive multinucleated cells with ≥ 3 nuclei were scored as osteoclasts using light microscopy [47]. To assess osteoclastic bone-resorptive function, OCPs were seeded onto dentin slices (IDS, AE-8050, United Kingdom) and cultured in the presence of 30 ng/mL M-CSF and 100 ng/mL RANKL for 10–11 days. Resorption lacunae formed on the dentin surface were visualized by Mayer's hematoxylin staining. Resorption pit areas were quantified via ImageJ.

### RNA sequencing

Total RNA was prepared using the RNeasy Mini kit (Qiagen, Germany). RNA-seq libraries were generated from 2 µg of total RNA using the SMARTer Stranded RNA-Seq Kit (TaKaRa Bio, United States). Paired-end sequencing (100 bp) was performed on an Illumina HiSeq 2500 system (Illumina, Inc., San Diego, United States). The resulting mRNA-seq datasets were processed as described previously [46, 48]. Briefly, raw reads were aligned to the mouse mm10 genome using HISAT2. Gene expression and DEGs were identified with HOMER using thresholds of ≥ twofold change and Benjamini–Hochberg-adjusted FDR < 0.05. K-means clustering and heatmap visualization were performed with the online Morpheus platform (https://software.boradinstitute.org/morpheus). DEGs were functionally annotated through GO enrichment analysis using Metascape. GSEA was conducted across the complete dataset of 1,731 genes using gene sets obtained from MSigDB. In addition, HOMER de novo motif analysis was also conducted to identify enriched transcription factor binding motifs.

### Real-time quantitative PCR

cDNA was synthesized using M-MLV reverse transcriptase (Promega, Madison, WI, USA), and quantitative PCR was performed using iQ SYBR Green Supermix on an iQ5 system (Bio-Rad, USA). Relative mRNA levels, normalized to β-actin, were determined using the primer sequences detailed in Supplementary Table 1.

### Quantification of intracellular ROS levels

To measure intracellular ROS, OCPs were cultured with RANKL (100 ng/mL) for 2 days, equilibrated in phenol red-free α-MEM for 1 h, and restimulated with RANKL for 10 min. Cells were then incubated with 0.5 μM CellROX™ Deep Red Reagent (Molecular Probes, USA) for 30 min prior to harvest [49]. Fluorescence was quantified at 660 nm using a CytoFLEX flow cytometer (Beckman Coulter, CA, USA), and data are presented as mean fluorescence intensity (MFI) from three independent experiments.

### Lentivirus-mediated RNA interference

For shRNA-mediated knockdown, shRNA-encoding DNA oligonucleotides targeting *Prdx1*, *Prdx2,* or *Traf6* were inserted into lentiviral vector pLKO.1 (Addgene). Lentivirus were produced by co-transfecting HEK 293 T cells with plasmids encoding VSVG, NLBH, and the specific shRNA constructs. To achieve target gene knockdown, OCP cells were transduced with lentiviruses and selected using 2 μg/ml puromycin and 120 ng/ml M-CSF for 2 days. Thereafter, selected OCP cells were utilized in subsequent experiments. The shRNA-encoding DNA oligonucleotides were described in Supplementary Table 1.

### Confocal microscopy

OCPs were seeded onto glass coverslips and stimulated with RANKL for 48 h. Cells were fixed in ice-cold methanol at − 20 °C for 10 min, washed with PBS, and blocked for 1 h at room temperature with 5% normal goat serum, 0.1% Triton X-100, and 1% BSA in PBS. Primary antibodies against PRDX1 (9D2) and LAMP1, diluted in PBS containing 1% BSA and 1% goat serum, were incubated overnight at 4 °C. Cells were then incubated with Alexa Fluor 488- or 568-conjugated secondary antibodies at room temperature. Nuclei were stained with DAPI. Images were obtained using an LSM900 confocal microscope (Carl Zeiss, Oberkochen, Germany).

### In vivo ubiquitylation assays

293 T cells were transfected with pcDNA3.1-Flag-Prdx1 (wild-type, K67A, C83S, Y194D, or Y194F mutant) or pcDNA3.1-Flag-Prdx2, together with HA-tagged ubiquitin (HA-Ub). After 24 h, cells were incubated with 1 mM H_2_O_2_ for 1 h. Cells were lysed in RIPA buffer [50 mM Tris–HCl (pH 7.4), 150 mM NaCl, 0.5% sodium deoxycholate, 0.1% SDS, and 1% NP-40]. Lysates were immunoprecipitated with an anti-FLAG antibody (Sigma, SAB4200071, USA), and precipitated proteins were examined by Western blotting using anti-HA–HRP and anti-FLAG antibodies. To assess TRAF6-mediated PRDX1 ubiquitylation, 293 T cells were transfected with pcDNA3.1-Flag-Prdx1 (wild-type or K67A), HA-tagged ubiquitin with or without pcDNA3.1-HisC-Traf6.

### Chromatin immunoprecipitation assay

ChIP assay was conducted according to methods detailed in previous studies [50]. Cells were fixed with 1% formaldehyde for 10 min and lysed in hypotonic buffer containing 10 mM HEPES–KOH (pH 7.8), 10 mM KCl, 1.5 mM MgCl₂, and protease inhibitors. Following centrifugation, the nuclear pellets were subjected to 20 cycles of sonication using a Bioruptor (Diagenode, London, UK), and ChIP was performed with anti-p65 or control IgG antibodies [51]. Immunoprecipitated DNA was analyzed by quantitative PCR using primers targeting the *Dcstamp, Mmp9*, and *Nfatc1* promoter regions. Primer sequences were provided in Supplementary Table 1.

### Statistical analysis

Data are presented as mean ± SD or mean ± SEM, as specified in the figure legends. Statistical significance was assessed using two-tailed t-tests, one-way ANOVA, or two-way ANOVA followed by Dunnett's, Šídák's, or Tukey's multiple-comparison tests, as appropriate. Sample sizes are presented in the figure legends. ns, not significant; *P < 0.05; **P < 0.01; ***P < 0.001; ****P < 0.0001. Data analysis was performed using GraphPad Prism 10 (GraphPad Software Inc., La Jolla, CA, USA). Each experiment was performed with at least two biological replicates to ensure consistency and reproducibility of the data.

## Supplementary Information


Supplementary Material 1.

## Data Availability

All data are included in the methods and supporting information of this article. RNA-seq data generated during this study are available under GEO accession number GSE248994.
