# Supplementary material for: A peroxiredoxin 1–reactive oxygen species feedback loop modulates nuclear factor kappa B activity in osteoclastogenesis
Source: Mol Biomed. 2026 Aug 27;7:154. doi: 10.1186/s43556-026-00559-5 (PMC13522327; doi:10.1186/s43556-026-00559-5)
Supplement: Supplementary file 1 [file 43556_2026_559_MOESM1_ESM.docx]

**A peroxiredoxin 1–reactive oxygen species feedback loop modulates**

**nuclear factor kappa B activity in osteoclastogenesis**

Hyerim Lee ^1^, Sheunghun Lee ^1^, You-Jee Jang ^2^, Kyubin Lee^1^, Seon Young Hwang^1^, Goo Taeg Oh^3^, Jae-Il Park ^4^, Sun-Ju Yi^1, *^, and Kyunghwan Kim^1,*^

^1^Department of Biology, Chungbuk National University, Cheongju, Chungbuk, Republic of Korea

^2^Department of Biomedical Laboratory Science, Honam University, Gwangju, Republic of Korea

^3^Department of Life Sciences, Ewha Womans University, Seoul, Republic of Korea

^4^Korea Basic Science Institute, Gwangju, Republic of Korea

^*^ Corresponding authors: Kyunghwan Kim, [kyungkim@chungbuk.ac.kr](mailto:kyungkim@chungbuk.ac.kr); Sun-Ju Yi, [sjyi@cbnu.ac.kr](mailto:sjyi@cbnu.ac.kr)

Keywords: PRDX1, reactive oxygen species, p65, osteoclastogenesis, osteoporosis

**Fig. S1. Related to Figure 1**

**a** Validation of *Prdx1* and *Prdx2* knockdown. Relative mRNA expression levels in OCPs transduced with control shRNA, *Prdx1* shRNA, or *Prdx2* shRNA. **b,c** Expression levels of Prdx1 and Prdx2 following Prdx1 knockdown. Relative mRNA expression levels were analyzed by RT-qPCR (b), and protein expression levels were examined by Western blotting (c). **d** Relative mRNA expression levels of other Prdx family members (*Prdx3*–*Prdx6*) in control and -depleted OCPs. Two-tailed *t*-test (a,b,d). *P < 0.05; **P < 0.01; ***P < 0.001; ****P < 0.0001.

**Fig. S2. Related to Figure 2**

Western blot analysis of PRDX1 in OCP cell lysates from WT mice (*Prdx1^+/+^*) or KO mice (*Prdx1^-/-^*).

**Fig. S3. Related to Figure 5**

Sequence alignment of mouse PRDX1 and PRDX2 proteins. Asterisks indicate residues putatively involved in ubiquitylation (K) and oxidation (C), which are present in PRDX1 but absent in PRDX2.

**Fig. S4. Related to Figure 6**

**a** TRAP staining of OCP cells expressing control shRNA or *Traf6* shRNAs. Scale bar, 200 μm **b,c** 293T cells were transfected with HA-*Prdx1* wild-type (WT), Y194D (YD), or Y194F (YF) together with Flag-*Traf6*. Cell lysates were immunoprecipitated with anti-Flag antibody and analyzed by Western blotting using anti-HA antibody to assess the interaction between PRDX1 and TRAF6. Expression of PRDX1 and TRAF6 was confirmed by immunoblotting with anti-HA and anti-Flag antibodies, respectively.

**Fig. S5. Related to Discussion**

**a** 293T cells transfected with Flag-*Prdx1* wild type or Flag-*Prdx1*-C83S along with HA-Ub were treated with 1 mM H₂O₂ for 1 h. Cell lysates were immunoprecipitated with anti-Flag antibody, and immunocomplex were separated and detected with western analysis using anti-HA and anti-Flag antibody. **b** 293T cells transfected with Flag-*Traf6* and HA-*Prdx1* were treated with 1 mM H_2_O_2_ for 30 min. Cell lysates were immunoprecipitated with an anti-Flag antibody, and the bound proteins were analyzed by Western blotting with anti-HA and anti-Flag antibodies.

**Supplementary Materials and method**

**Antibodies and reagents**

The antibodies used in this study were listed below: antibodies for p-ERK, ERK, p-p65(S536), p65, and p-PRDX1 (Y194) were purchased from Cell Signaling Technology (Danvers, MA, USA); antibodies for p-p38, p38, p-JNK, and JNK were purchased from Millipore (Burlington, MA, USA); antibodies for Lamin A/C, β-Actin, and PRDX1 (9D2) were obtained from Santa Cruz Biotechnology (Santa Cruz, CA, USA); antibodies for FLAG and HA were purchased from sigma-Aldrich (St. Louis, MO, USA); antibody for Xpress was purchased from Invitrogen (Carlsbad, CA, USA); antibodies for PRDX1, TRAF6, and LAMP1 were from Abcam (London, UK); an p65 antibody for ChIP was obtained from Diagenode (Denvile, NJ, USA); an PRDX2 antibody was generously provided by Dr. Jae-Il Park. All other reagents were purchased from Sigma Aldrich (St. Louis, MO, USA).

**Whole cell lysates and Subcellular fractionation**

Whole-cell protein extracts were generated using a conventional lysis buffer supplemented with protease inhibitors, 1 mM sodium orthovanadate, and 2.5 mM sodium pyrophosphate. Equal amounts of protein were resolved by SDS–PAGE, transferred to membranes, and immunoblotted with the indicated primary antibodies. For subcellular fractionation, cells were incubated on ice for 8 min in buffer A [10 mM HEPES (pH 7.9), 10 mM KCl, 1.5 mM MgCl₂, 0.34 M sucrose, 10% glycerol, 1 mM DTT, 0.1% Triton X-100, and protease inhibitors]. After centrifugation at 300 × g for 5 min at 4°C, the supernatant was collected as the cytosolic fraction. The nuclear pellet was washed, lysed in SDS buffer, and briefly sonicated. Both fractions were analyzed by Western blotting with the indicated antibodies.

**Supplementary Table 1. Primers**

| **Target** | **Forward sequences (5’ → 3’)** | **Reverse sequences (5’ → 3’)** |
| --- | --- | --- |
| **RT-PCR** | | |
| *Actin* | GCAAGTGCTTCTAGGCGGAC | AAGAAAGGGTGTAAAACGCAGC |
| *Dcstamp* | CCGCTGTGGACTATCTGCTG | CTCAATGGCTGCTTTGATCG |
| *Idh2* | CCGTCTTCAGAGAGCCAATC | GAAATGGACTCGTCGGTGTT |
| *Mmp9* | CGTCGTGATCCCCACTTACT | AACACACAGGGTTTGCCTTC |
| *Nfatc1* | CTCGAAAGACAGCACTGGAGCAT | CGGCTGCCTTCCGTCTCATAG |
| *p65* | GGAGTTCCAGTACTTGCC | GTCCTTTTGCGCTTCTCT |
| *Prdx1* | CACCCAAGAAACAAGGAGGA | AAAAAGGCCCCTGAAAGAGA |
| *Prdx2* | GGTGCCTTCAAGGAAATCAA | GCCTAGCTTTCGGAAGTCCT |
| *Scd2* | GCTCTCGGGAGAACATCTTG | CAGCCCTGGACACTCTCTTC |
| *Traf6* | AAACCACGAAGAGGTCATGG | GCGGGTAGAGACTTCACAGC |
| **shRNA** | | |
| *Prdx1* sh | CCGGCCCATGAACATTCCCTTAATACTCGAGTATTAAGGGAATGTTCATGGGTTTTTG | AATTCAAAAACCCATGAACATTCCCTTAATACTCGAGTATTAAGGGAATGTTCATGGG |
| *Prdx2* sh | CCGGCTGCTTGCTGACGTGACTAAACTCGAGTTTAGTCACGTCAGCAAGCAGTTTTTG | AATTCAAAAACTGCTTGCTGACGTGACTAAACTCGAGTTTAGTCACGTCAGCAAGCAG |
| *Traf6* sh1 | CCGGCCCAGGCTGTTCATAATGTTACTCGAGTAACATTATGAACAGCCTGGGTTTTTG | AATTCAAAAACCCAGGCTGTTCATAATGTTACTCGAGTAACATTATGAACAGCCTGGG |
| *Traf6* sh2 | CCGGGCCTGCATCATCAAATCCATACTCGAGTATGGATTTGATGATGCAGGCTTTTTG | AATTCAAAAAGCCTGCATCATCAAATCCATACTCGAGTATGGATTTGATGATGCAGGC |
| **ChIP** | | |
| *Dcstamp (-0.6kb)* | TTTCAAATGCTATCCCCAAA | GGCTTAGTCGGCCATCATT |
| *Mmp9 (-0.5kb)* | TCTTTCCTTCCCCAAGGAGT | CCATCCCCACACTGTAGGTT |
| *Nfatc1 (-0.7kb)* | CTCGTACAGCAAGCAATCCA | ATGTAAAATCGCAGGCTTCC |
